# Utilization of Receptor-Binding Domain of SARS-CoV-2 Spike Protein Expressed in *Escherichia coli* for the Development of Neutralizing Antibody Assay

**DOI:** 10.1007/s12033-022-00563-4

**Published:** 2022-09-14

**Authors:** Termsak Tantiwiwat, Apisitt Thaiprayoon, Ake-kavitch Siriatcharanon, Chakrit Tachaapaikoon, Nongluk Plongthongkum, Dujduan Waraho-Zhmayev

**Affiliations:** 1 Biological Engineering Program, Faculty of Engineering, King Mongkut’s University of Technology Thonburi, Bangkok, 10140 Thailand; 2 School of Bioresources and Technology, King Mongkut’s University of Technology Thonburi, Bangkok, 10150 Thailand; 3 Excellent Center of Enzyme Technology and Microbial Utilization, Pilot Plant Development and Training Institute, King Mongkut’s University of Technology Thonburi, Bangkok, 10150 Thailand

**Keywords:** Receptor-binding domain (RBD), SARS-CoV-2, Neutralizing antibodies, sVNT, ELISA, *Escherichia coli*

## Abstract

The ongoing COVID-19 pandemic has resulted from widespread infection by the SARS-CoV-2 virus. As new variants of concern continue to emerge, understanding the correlation between the level of neutralizing antibodies (NAb) and clinical protection from SAR-CoV-2 infection could be critical in planning the next steps in COVID-19 vaccine programs. This study explored the potential usefulness of *E. coli* as an alternative expression system that can be used to produce a SARS-CoV-2 receptor-binding domain (RBD) for the development of an affordable and flexible NAb detection assay. We expressed the RBD of Beta, Delta, and Omicron variants in the *E. coli* BL21(DE3) strain and purified them from whole bacterial cells using His-tag-mediated affinity chromatography and urea-assisted refolding. Next, we conducted a head-to-head comparison of the binding activity of our *E. coli*-produced RBD (E-RBD) with commercial HEK293-produced RBD (H-RBD). The results of a direct binding assay revealed E-RBD and H-RBD binding with ACE2-hFc in similar signal strengths. Furthermore, in the NAb detection assay, % inhibition obtained from both E-RBD and H-RBD demonstrated comparable results in all the investigated assays, suggesting that non-glycosylated RBD produced from *E. coli* may offer a cost-effective alternative to the use of more expensive glycosylated RBD produced from human cells in the development of such an assay.

**Supplementary Information:**

The online version contains supplementary material available at 10.1007/s12033-022-00563-4.

## Introduction

The COVID-19 pandemic has imposed a devastating impact on humanity in many ways, spurring the employment of global joint efforts to combat this devastating infectious disease. Interaction of the SARS-CoV-2 spike receptor-binding domain (RBD) with the angiotensin-converting enzyme 2 (ACE2) receptor on the host’s cell surface is essential for viral entry [1]. An early study performed on 149 convalescent plasma samples collected from recovered COVID-19 patients prior to the availability of a COVID-19 vaccine reported that RBD-specific antibodies with potent antiviral activity were observed in all individuals tested [2]. This study was in line with previous studies showing that neutralizing antibodies (NAbs) of SARS-CoV, another deadly coronavirus that caused an epidemic outbreak in 2003, could bind the spike RBD and block ACE2 binding [3, 4]. Accordingly, RBD is the main target for designing vaccines and therapeutics

In addition to vaccines and therapeutics, diagnostics represents another area that plays a vital role in the management of COVID-19. By analyzing data from vaccinated and convalescent individuals, Khoury et al. [5] confirmed that NAb levels are highly predictive of immune protection from symptomatic SARS-CoV-2 infection [5]. The gold standard method for measuring the levels of NAb in serum and plasma for many viral diseases is the plaque reduction neutralization test (PRNT). In the case of SARS-CoV-2, this technique uses live virus, which mandates many limitations, especially the requirement of a specialized biosafety level 3 (BSL-3) containment facility [6]. Hence, several techniques have been developed for the detection of NAb in ELISA formats that mimic the interaction between RBD and ACE2 [7–9]. These assay formats are simpler, faster, less labor-intensive, and—importantly—can be performed in a BSL-2-level facility. Tan et al.’s [9] surrogate virus neutralization test (sVNT) technique is now commercially available under the trade name cPass™ SARS-CoV-2 Neutralization Antibody Detection Kit and has been issued Emergency Use Authorization (EUA) by The United States Food and Drug Administration (U.S. FDA). The cPass kit has also acquired CE marking in Europe and is authorized for use in other countries, such as Brazil, Singapore, and the United Arab Emirates [10]. Although these testing approaches may be affordable in high-income countries, implementing them in low- and middle-income countries may face obstacles. Therefore, the development of low-cost rapid diagnostic kits will be beneficial in facilitating worldwide data collection.

The SARS-CoV-2 spike (S) protein is a glycosylated protein containing disulfide bonds. Currently, the spike RBD is prevalently expressed in the eukaryotic cell expression system Chinese hamster ovarian cell line (CHO), the human embryonic kidney 293 cell line (HEK293), and baculovirus-infected insect cell cultures [11–13]. However, employing the eukaryotic expression system also entails various drawbacks, such as the difficulty of the culture procedure, high cost, extensive labor, and the long time required for stable cell line development. As the disease continues to spread and new variants of concern (VoC) repeatedly emerge, the production of RBD variants from microorganisms such as *E. coli* offers an alternative system that promises flexibility, cost-effectiveness, and fast and simple production [14]. Several previous investigations have demonstrated the capability of using *E. coli* as a host for RBD production [15–18]. Specifically, Fitzgerald et al. [16] evaluated the direct ELISA assay for the detection of RBD binding antibodies from human sera using the His-tag S319-640 fragment containing RBD produced in *E. coli* in comparison to the S319-591 RBD containing fragment expressed in human cell lines [16]. Márquez-Ipiña et al. [18] compared the binding of human sera to RBD (N318-V510) produced in *E. coli* or a commercial full-length S protein in direct and sandwich ELISA formats [18]. According to the study findings, even though the bacterial-produced RBD lacked glycosylation, RBD fragments produced in *E. coli* could still be recognized by antibodies, which may support the production of a diagnostics kit.

In this research, we constructed a series of pET28-RBD plasmids using RBD genes of Alpha (B.1.17), Beta (B.1.351), Delta (B.1.617.2), and Omicron (B.1.1.529) variants with *E. coli* codon optimization. Using the Delta variant as a model system, we assessed if common B strain derivatives of *E. coli*, specifically BL21 (DE3) and SHuffle® T7 express (hereafter SHuffle), could express RBDs that can bind to ACE2. Thus, the soluble and insoluble expression of RBD of the Delta variant in these strains under varying experimental conditions were analyzed as a model system, and the most suitable condition was applied for other variants. Even though RBD could be expressed in both forms, most of the protein found occurred in insoluble form. Therefore, we purified the RBD variants from whole cells and evaluated the binding activity of our *E. coli*-produced RBD compared with commercial HEK293-produced RBD using direct binding with the ACE2 receptor and competitive ELISA assays. According to our findings, the competitive ELISA technique demonstrated in this study can be applied to the development of a NAb detection assay. To the best of our knowledge, no previous study has conducted a head-to-head comparison of 3 RBD variants, Beta, Delta, and Omicron, produced in *E. coli* and in HEK293 cells for such an assay.

## Materials and Methods

### Bacterial Strains and Construction of E-RBD Expression Vectors

The *E. coli* BL21(DE3) and SHuffle® T7 express strains were obtained from New England Biolabs (NEB). The DNA sequence of SARS-CoV-2 spike RBD (amino acid R319–F541 of S protein) from the Beta, Delta and Omicron variants were codon-optimized for *E. coli* expression and synthesized by GenScript with *Nco*I and *Sal*I flanking restriction sites. The RBD genes were digested and cloned into the pET28 expression vector resulting in the recombinant protein expressed with C-terminal 6xHis-tag. The protein sequence of RBD variants were based on wild-type SARS-CoV-2 (YP_009724390.1) with the following mutations: Alpha (B.1.17) variant with N501Y mutation; Beta (B.1.351) variant with K417N, E484K, and N501Y mutations; Delta (B.1.617.2) variant with L452R and T478K mutations; and Omicron (B.1.1.529) variant with G339D, S371L, S373P, S375F, K417N, N440K, G446S, S477N, T478K, E484A, Q493R, G496S, Q498R, N501Y, and Y505H mutations.

### Protein Expression for Optimal Condition Identification

The overnight culture of the *E. coli* BL21(DE3) and SHuffle strains containing pET28-RBD plasmids were subcultured (1% v/v) into 100 mL of fresh Luria–Bertani (LB) medium in 250 mL shake flasks and grow at 37 °C, 200 RPM until OD_600_ reached 0.6 – 0.7. Then each culture was induced with IPTG, and protein expression was allowed to continue under different conditions (i.e., at 25, 30, or 37 °C and at 0, 0.1, or 0.5 mM IPTG for another 2 or 4 h).

### SDS-PAGE Analysis

For optimal condition identification, we prepared soluble and insoluble fractions as described. The samples were normalized to OD_600_ = 25 before harvesting. Cell pellets were resuspended with 300 µL phosphate-buffered saline (PBS) before sonicating and centrifuging at 10,000 x*g* for 10 min. The supernatant was collected as the soluble fraction of the whole-cell lysate. The pellet part was washed 2 times with 500 µL TE buffer (50 mM Tris–HCl pH 8.0 and 1 mM EDTA), resuspended with PBS containing 2% w/v SDS and boiled at 100 °C for 5 min then centrifuged at 10,000 x*g* for 10 min. The supernatant was collected as the insoluble fraction. Samples were mixed with 6 × gel loading dye (Bio-Rad) containing 10% v/v β-mercaptoethanol, then boiled at 100 °C for 5 min. Next, 40 µL of samples were loaded onto 10% polyacrylamide gels that were prepared according to the manufacturer’s protocol (TGX FastCast Acrylamide Solutions; Bio-Rad). Protein staining was performed using InstantBlue® Coomassie Protein Stain (Abcam). Gels were soaked overnight and were washed the next day with washing buffer (10% v/v acetic acid mixed with 10% v/v methanol). Gel images were captured by Gel Doc EZ Gel Documentation System (Bio-Rad). We ensured purified protein samples by quantifying all samples using the Bradford assay, and 0.5 µg of each fraction was loaded per well.

### Refolding and Purification of E-RBD

Purification was accomplished by subculturing *E. coli* BL21(DE3) cells into 200 mL of fresh LB medium in 1-L shake flasks and protein expression was performed at 37 °C with 0.1 mM IPTG for 2 h. Cells were harvested from 200 mL culture and resuspended (1 g of cells wet weight per 4 mL) in Base buffer (20 mM Tris–HCl, 6 M urea, 500 mM NaCl, 5 mM β-mercaptoethanol and 10% v/v glycerol) and sonicated on ice using Sonifier® SFX150 cell disruptors and homogenizers (Branson) 70 times at 30 s intervals with 45% amplitude, 50% duty cycle. After sonication, refolding buffer (20 mM Tris–HCl, 500 mM NaCl, 55 mM glucose, 2 mM reduced glutathione (GSH), 0.2 mM oxidized glutathione (GSSG) and 20% v/v glycerol) supplemented with 2 M urea was added to the lysate at 1:9 (v/v) ratio, and incubated a on rocking shaker for 1 h at 20 °C. The precipitant in solution was then removed by centrifuging at 4 °C, 10,000 x*g* for 30 min. The supernatant solution was applied to a 5-mL HisTrap column (GE Healthcare) at a flow rate of 1 mL/minute at room temperature. Protein trapped on the HisTrap column was refolded with 2 CV of a series of refolding buffers containing 1.5, 1, 0.5 and 0 M urea. Non-specific proteins were washed off from the HisTrap column with 10 CV of refolding buffer containing 30 mM imidazole and 10 CV of the same buffer containing 50 mM imidazole. The target protein was eluted with 5 CV of the same buffer containing 70, 100, and 500 mM imidazole. Finally, imidazole was removed from the sample using 10 kDa molecular weight cut-offs in a centrifugal concentrator while performing buffer exchange with Tris-buffered saline (TBS).

### Western Blot Analysis

Western Blot analysis was performed using a standard protocol. Briefly, protein separation was performed in 10% TGX gel, transferred to a PVDF membrane (Bio-Rad) using the Trans-Blot Turbo Transfer System (Bio-Rad), and blocked overnight with 5% w/v nonfat dry milk in tris-buffered saline buffer containing 0.1% Tween-20 (TBST). Membranes were washed with TBST and probed with HRP-conjugated rabbit polyclonal antibody to 6X His-tag® (1:5,000; Abcam) or rabbit polyclonal antibody to SARS-CoV-2 spike RBD (1:3,000; Sino Biological) with HRP-conjugated goat polyclonal secondary antibody to rabbit IgG-Fc (1:30,000; Sino Biological). Digital imaging entailed using Clarity™ Western Enhanced chemiluminescence (ECL) Substrate and Clarity Western Luminol/Enhancer Reagent (Bio-Rad) and the images were captured using the ChemiDoc™ MP Imaging System (Bio-Rad).

### Direct Binding and NAb Detection Assays

ELISA assays were performed following the general ELISA protocol from Bio-Rad. A SpectraPlate™-96 HB (PerkinElmer) was coated overnight at 4 °C with human ACE2-hFc (Sino Biological) at 130 ng per well in 50 μL of 50 mM carbonate-bicarbonate buffer (pH 9.6). The plate was washed 3 times with PBS containing 0.05% v/v Tween-20 (PBST), blocked with 300 μL 1% w/v BSA in PBS and incubated for 1 h at 37 °C. For the direct binding assay, 50 μL of PBST containing 0, 2, 4, 6, 8 and 10 nM of E-RBD or H-RBD and incubated for 90 min at 37 °C. After washing again, 50 μL of primary antibody diluted in PBST was added to each well and incubated for 1 h at 37 °C. After washing, 50 μL of HRP-conjugated secondary antibody diluted in PBST was added to each well and incubated for another 1 h at 37 °C. After final washes, 50 μL of 3,3’,5,5’-tetramethylbenzidine (TMB) (Abcam Inc.), a chromogenic substrate for HRP was added to each well and incubated at room temperature in the dark for 15 min. Finally, 50 μL of stop solution (1 N HCl) was added and the absorbance was measured at 450 nm (Infinite M200, Tecan Austria GmbH, Grödig Austria). For the NAb detection assay, we performed a competitive ELISA. After a blocking step, a mixture of 50 μL of PBST containing 3 ng of E-RBD or H-RBD was pre-incubated with SARS-CoV-2 spike neutralizing antibody at 20,000, 4,000, 800, 160, 32, 6.4, 1.28, 0.256, 0.0512 and 0 ng/mL at 37 °C for 1 h.

For both assays, when HRP-conjugated rabbit polyclonal antibody to 6X His-tag (1:5,000; Abcam Inc.) was used as the primary antibody, no secondary antibody was used. However, when rabbit polyclonal antibody to SARS-CoV-2 spike RBD (1:5,000; Sino Biological) was used as the primary antibody, then HRP-conjugated goat polyclonal secondary antibody to rabbit IgG-Fc (1:10,000; Sino Biological) was required as a secondary antibody. SARS-CoV-2 spike neutralizing antibodies used in the neutralizing detection assay were SARS-CoV-2 (2019-nCoV) spike neutralizing antibody, mouse Mab (Cat. No. 40591-MM43) and SARS-CoV-2 (2019-nCoV) spike neutralizing antibody, and rabbit Mab (Cat. No. 40592-R0004 and 40592-R117) (Sino Biological). The following commercial H-RBDs from Sino Biological were used: Beta (B.1.351) variant (Cat. No. 40592-V08H85); Delta (B.1.617.2) variant (Cat. No. 40592-V08H90); and Omicron (B.1.1.529) variant (Cat. No. 40592-V08H121). ACE2-hFc (Cat. No. 10108-H02H) was also purchased from Sino Biological.

The % inhibition was calculated using this equation: Inhibition (%) = (1 − sample optical density value/negative control optical density value) × 100.

### 3D Structural Analysis

The 3D structure modeling of the variant RBDs was performed by the automated protein structure homology-modelling server (SWISS-MODEL; https://swissmodel.expasy.org/interactive). All of the image models were illustrated using PyMOL.

## Results

### The Expression of Recombinant RBD in *E. coli* BL21(DE3) and Shuffle Strains

**Fig. 1:**
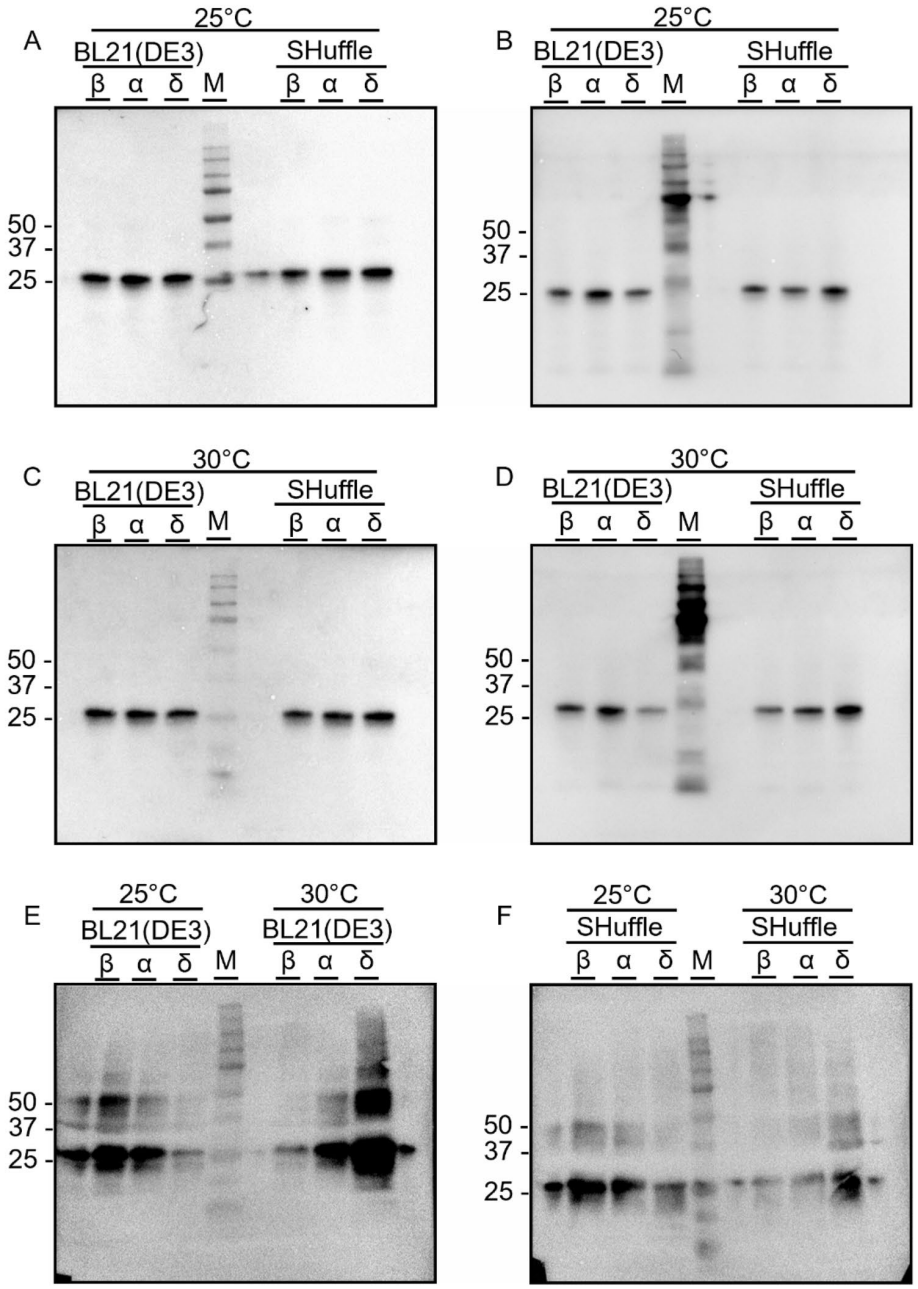
The WB analysis of the recombinant RBD produced from *E. coli* BL21(DE3) and SHuffle strains with different detection antibodies. The RBD-His in soluble fractions expressed at (**A**) 25 °C and (**C**) 30 °C were detected by anti-SARS-CoV-2 spike RBD antibody and at (**B**) 25 °C and (**D**) 30 °C were detected by anti-His-tag antibody. Insoluble RBD proteins (**E**) expressed in BL21(DE3) and (**F**) in SHuffle at 25 °C and 30 °C C were detected by anti-His-tag antibody

**Fig. 2:**
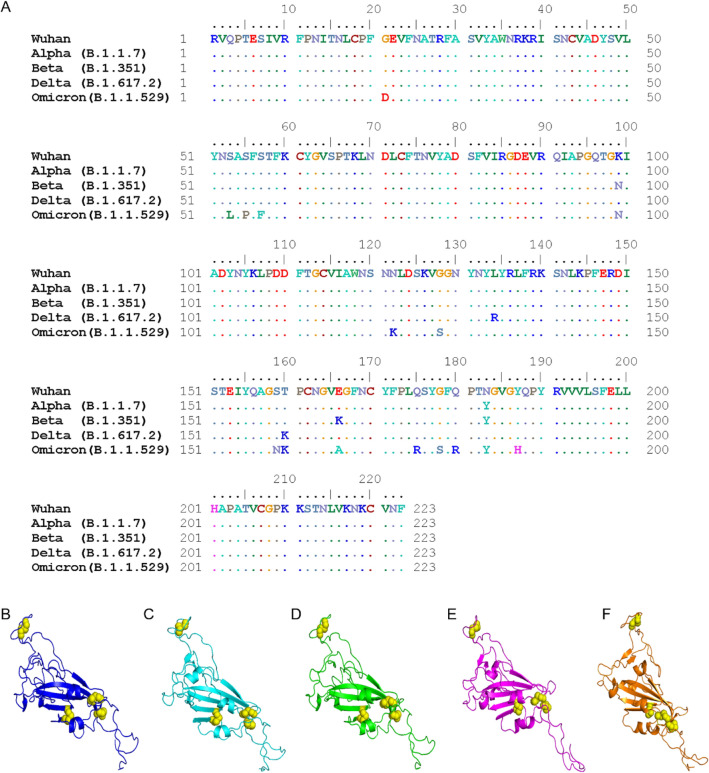
Comparisons of amino acid residues and the 3D structure of RBD variants. **A** The amino acid residue alignment of the RBD among the VoC (Beta, Delta, and Omicron variants) compared with the original strain (Wuhan). The 3D structure of **B** Wuhan RBD compared with **C** Alpha, **D** Beta, **E** Delta and **F** Omicron variants

The S protein of the coronavirus is known to be susceptible to a reducing environment. There are 4 disulfide bonds presenting within the amino acid residue 319–541 of the RBD domain, which can lead to protein misfolding [19]. In our chosen approach to address this issue, we used the *E. coli* SHuffle strain because it was engineered to possess an oxidized cytoplasmic environment that supports disulfide bond formation [20] while using BL21(DE3) to represent the common expression strain in the laboratory. After expression, we performed Western blot (WB) analysis using both rabbit polyclonal antibody anti-SARS-CoV-2 spike RBD (hereafter anti-spike RBD) *(Fig. 11A, C, and E ) and rabbit polyclonal 6X His-tag® antibody conjugated to HRP (hereafter anti-His) *(Fig. 1B, D, and F ) to confirm the expression of the recombinant RBD at 25 kDa. The results revealed that both strains could produce all RBD variants with comparable soluble protein (Fig. 1A–D), while the insoluble protein bands showed some variation depending on the variant and the host strain (Fig. 1E–F). All variants of the soluble recombinant RBD fraction revealed a single band at the predicted size. However, the insoluble fractions from both BL21(DE3) and Shuffle strains also exhibited the RBD bands and some aggregation. As expected, Shuffle yielded a lower amount of insoluble protein, as this strain was engineered for disulfide-bonded protein production. Further investigation was performed using Delta RBD as a representative for the optimization of expression conditions. The optimal conditions obtained were then used for other variants since the amino acid residues were quite similar; therefore, they would not be expected to affect the disulfide bond formation, as shown in the 3D structures (Fig. 2). It should be noted that in the later experiments, we included the Omicron variant, which eventually became a VoC and the major circulating variant worldwide.

**Fig. 3:**
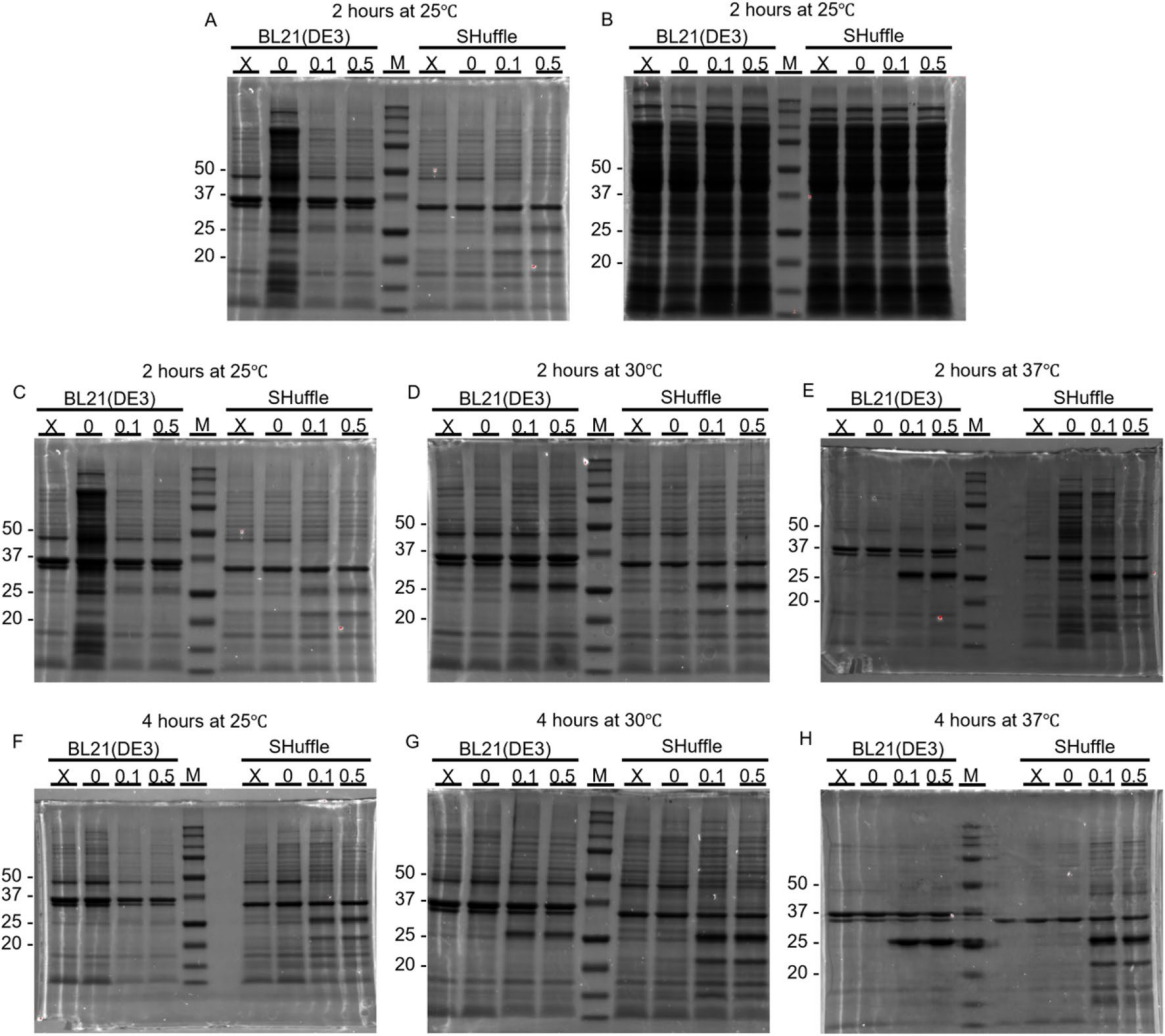
Coomassie staining of SDS-PAGE showing the bands that include recombinant RBD produced from *E. coli* BL21(DE3) and SHuffle strains. **A** Insoluble and **B** soluble fractions of samples were induced at 25 °C with various IPTG concentrations (0, 0.1, and 0.5 mM). X indicates fractions collected from *E. coli* without pET28 RBD plasmid. Insoluble protein induced for 2 h at **C** 25 °C, **D** 30 °C, and **E** 37 °C and 4 h at **F** 25 °C, **G** 30 °C, and **H** 37 °C with various IPTG concentrations are shown in the figure

The Coomassie-stained SDS-PAGE revealed that the recombinant Delta RBD was successfully expressed in soluble and insoluble fractions from both *E. coli* BL21(DE3) and SHuffle strains at 25 kDa, as shown in Fig. 3. Most of the RBD produced in both strains was found in inclusion bodies; specifically, it was clearly detected in the insoluble fractions (Fig. 3A) but was difficult to detect in the soluble fractions (Fig. 3B). Hence, the amount of RBD from the insoluble fractions of various conditions was quantitated using densitometry to obtain relative data per 100 mL cell culture volume (Supplementary data). All of the samples were normalized based on the band intensity of the reference condition (0.1 M IPTG, 25 °C, 2 h). The results displayed in Table S1 support the conclusion that because the IPTG induction level had only a slight effect on the RBD obtained from inclusion bodies, using a smaller amount of inducer would be more suitable as well as more cost-effective. At lower temperatures (25 and 30 °C), higher RBD could be obtained in the SHuffle strain; nevertheless, the amount was similar to that obtained from BL21(DE3) at 37 °C (Supplementary data). Consequently, we decided to purify RBD from a combination of both soluble and insoluble fractions from the BL21(DE3) strain. This decision was based on the determination that the SHuffle strain could not improve the RBD expression in the soluble fraction, while BL23(DE3) offered more advantages, including faster growth, the ability to scale up, and its status as a more commonly used strain in the laboratory. Therefore, expression in BL21(DE3) at 37 °C for 2 h with 0.1 mM IPTG was used for all RBD variants.

### Purification of SARS-CoV-2 Spike RBD from Bacterial Cells

**Fig. 4:**
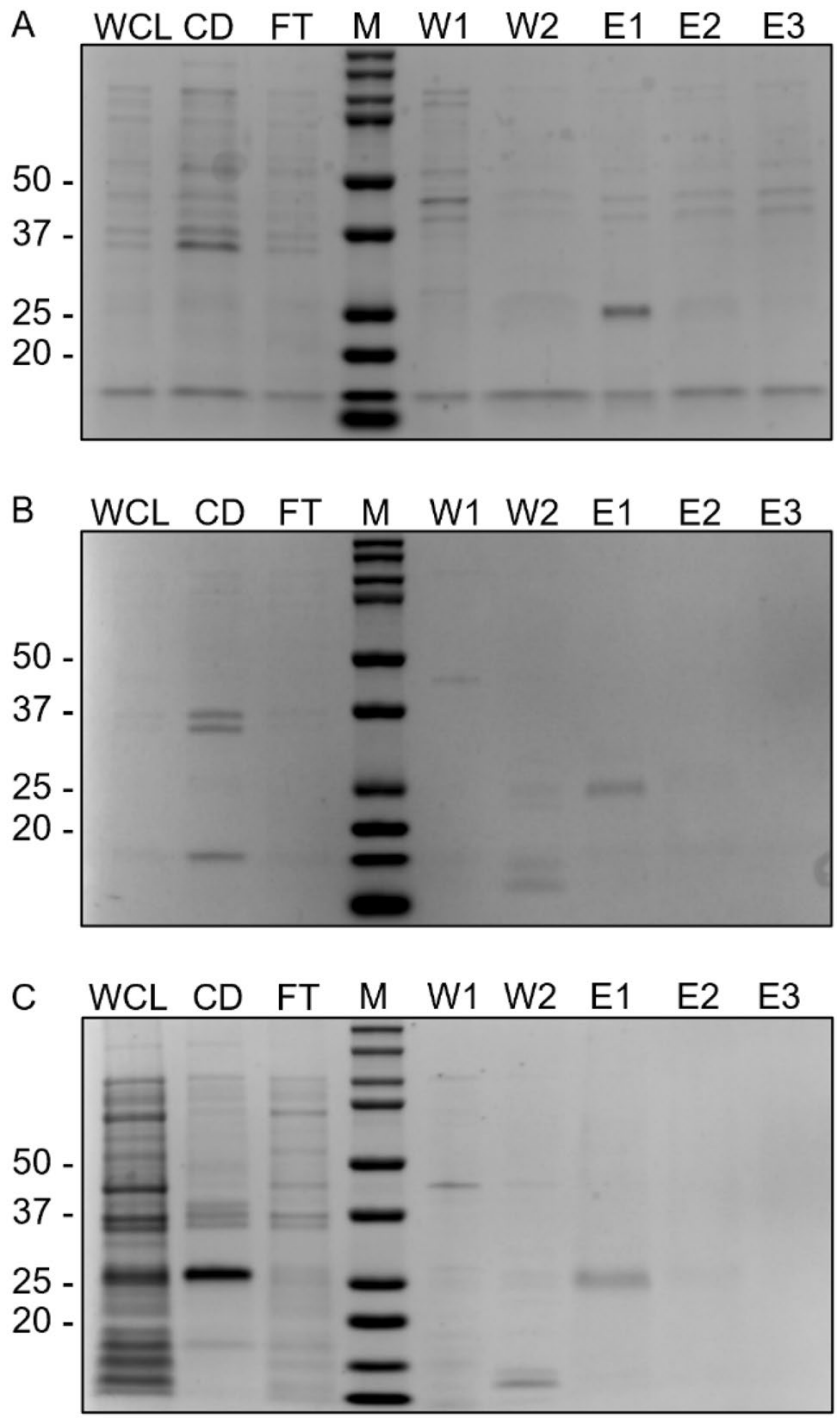
Coomassie staining of SDS-PAGE and densitometry analysis of purified RBDs from IMAC. **A** SARS-CoV-2 spike RBD protein of Beta, **B** Delta, and **C** Omicron variants were analyzed. Each gel contained whole-cell lysate (WCL), cell debris (CD), flow-through (FT), protein marker (M), wash (W1 and W2), and elution (E1–E3). E1 from each E-RBD sample shown here (Fig. 4A–C, Lane E1) was further analyzed with densitometry analysis for purity identification

SARS-CoV-2 spike RBD from the Beta, Delta, and Omicron variants were harvested from whole bacterial cells and purified by on-column refolding using the immobilized metal ion affinity chromatography (IMAC) method. Purified protein was analyzed with SDS-PAGE, as shown in Fig. 4. We observed that weakly bound protein and other contaminants were washed away with 10 column volumes (CV) of washing buffer containing 30 and 50 mM imidazole (Fig. 4, Lanes W1 and W2, respectively), while most of our target protein was successfully purified and eluted with washing buffer containing 70 mM imidazole (Fig. 4, Lane E1). The major band present in this fraction had an approximate size of 25 kDa, which corresponded to the predicted size of non-glycosylated RBD. However, some of our target protein was still observed in cell debris, flow-through, and elution fractions containing 100 and 500 mM imidazole (Fig. 4, CD, FT, Lanes E2 and E3, respectively). The results displayed in Fig. 4 were analyzed via Image Lab software (Bio-Rad) to detect the major protein bands present in Fig. 4, Lane E1, as well as by a densitometry technique. After background value subtraction and calculation with other detectable protein bands, the purity of the purified RBDs was approximately 70% for the Beta variant and 90% for the Delta and Omicron variants, resulting in product yield of E-RBD of 1, 0.825, and 0.4 mg/L from 200 mL culture volume in 1-L shake flasks.

**Fig. 5:**
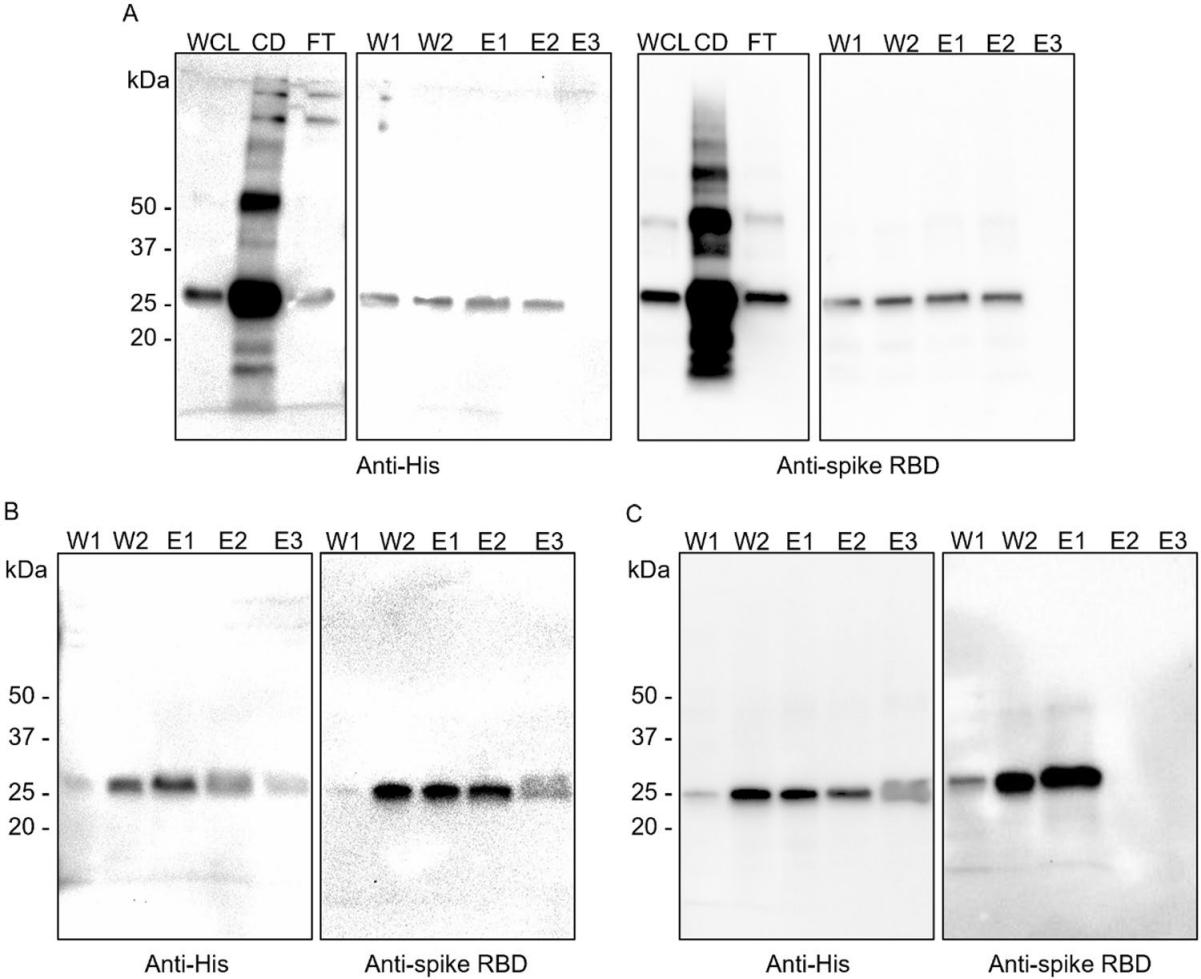
WB analysis of purified samples. **A** SARS-CoV-2 spike RBD proteins of Beta, **B** Delta, and **C** Omicron variants were detected with anti-His or anti-spike RBD as indicated

As several bands were observed in the Coomassie staining results, the samples were verified with WB under denaturing conditions using both anti-His and anti-spike RBD, as anti-spike RBD should be specific to the target protein. The results showed that the anti-His antibody was specific to our RBD-His recombinant protein: the bands detected from anti-His and anti-spike RBD were similar in each elution fraction (Fig. 5, Lanes W1, W2, E1—E3), while non-specific signals were mostly present in whole-cell lysate, cell debris, and the flow-through fraction (Fig. 5, Lanes WCL, CD, and FT). Both Coomassie staining and WB indicated that the target protein was still present in the discarded cell debris fraction (Fig. 5, Lane CD).

### Comparisons of Binding Between Non-Glycosylated E-RBD or Glycosylated H-RBD to ACE2 Using ELISA Assay

**Fig. 6:**
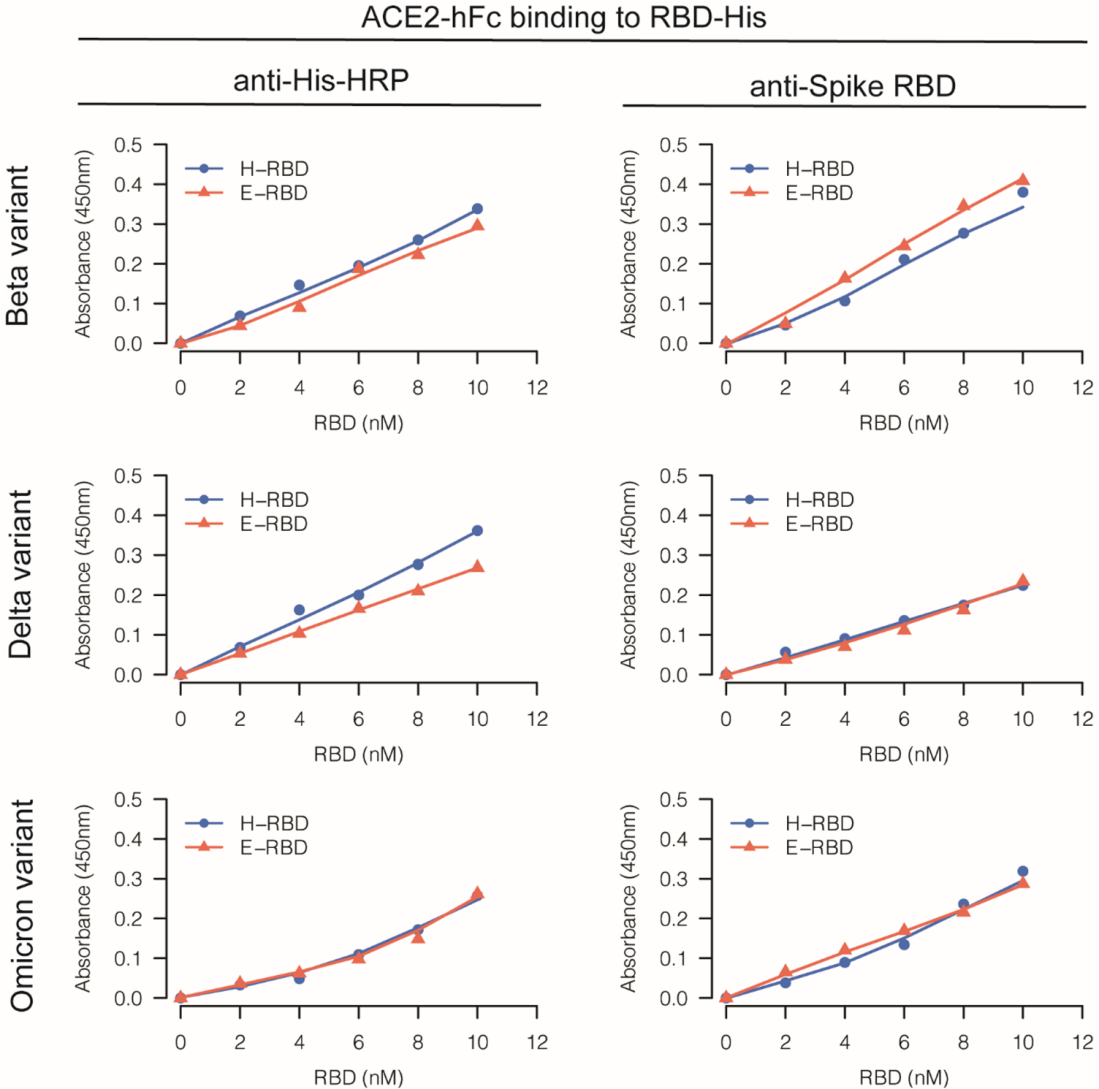
Comparison of ELISA signals detected from direct binding assay between E-RBD or H-RBD with ACE2-hFc. The left panel illustrates the signals obtained from anti-His HRP while the right panel illustrates the signals obtained from anti-spike RBD. The data represent the average of technical triplicate samples for both E-RBD and H-RBD

Because RBD produced from bacteria lacks glycosylation, it was crucial to verify whether glycosylation could affect the binding between RBD and ACE2 in an in vitro assay such as ELISA. We verified this potential effect using RBD containing C-terminal His-tags from 3 SARS-CoV-2 variants produced in-house and compared with commercial RBD-His produced from HEK293 cells. In this study, 130 ng of ACE2-hFc (equivalent to 100 ng ACE2 that was used in Tan et al. [9]) was coated per well and increasing concentrations of RBD between 0 and 10 nM were added. Both anti-His and anti-spike RBD antibodies were used for ELISA signal comparison. A dose-dependent specific binding between ACE2 and E-RBD or H-RBD was observed. Interestingly, in this direct binding assay, E-RBD and H-RBD demonstrated binding with ACE2-hFc with a very comparable signal strength (Fig. 6).

### Design of the NAb Detection Assay

Many studies performed on human serum have shown that NAb detection assays using competitive ELISA based on RBD and ACE2 interaction correlated well with other types of neutralization assays that require live viruses [7–9]. Byrnes et al. [8] developed an assay based on the adsorption of RBD moiety in various formats onto the surface of an ELISA plate, and ACE2-Fc was added to the sample [8]. In contrast, Tan et al. [9] coated ACE2 onto the surface of an ELISA plate, and RBD-HRP was added to the sample [9].

**Fig. 7:**
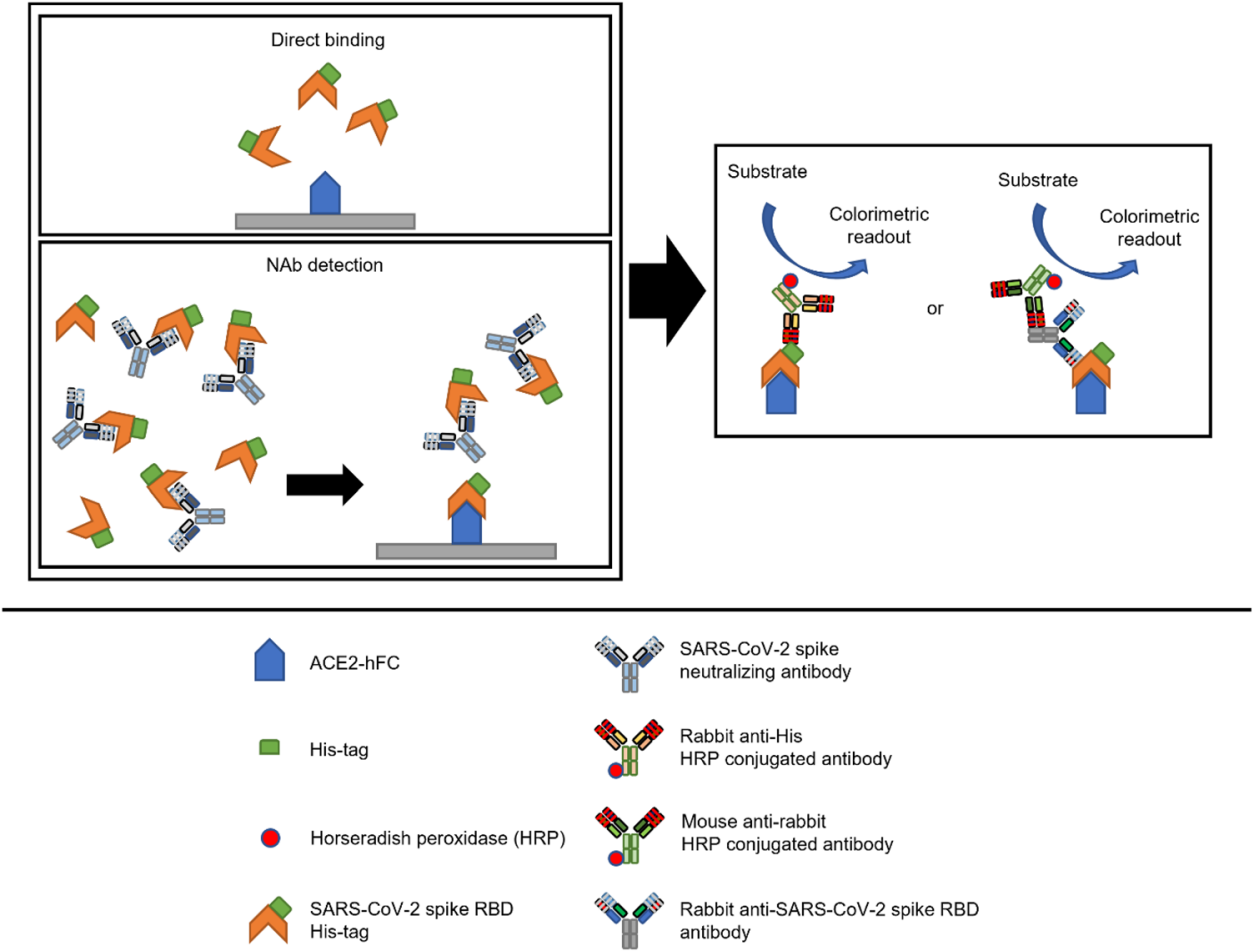
Schematic of NAb detection assay based on competitive ELISA format and detection strategy using anti-His HRP or anti-spike RBD antibodies as depicted

For the purposes of our study, we wanted to create an assay that could be flexible in terms of RBD variants used in the assay; therefore, our design entailed coating the ACE2-hFc on the surface. This method is similar to the method developed by Tan et al. (2020); however, we used the disulfide-linked homodimeric ACE2-hFc fusion protein instead of monomeric ACE2, as the structural analysis suggested that full-length ACE2 assembled into a dimeric structure, resulting in the simultaneous binding of two S protein trimers to an ACE2 dimer [21]. In our view, this configuration may resemble the native interaction more closely. Figure 7 offers a schematic illustration of our assay.

### Comparison of E-RBD and H-RBD for the Development of NAb Detection Assay

**Fig. 8:**
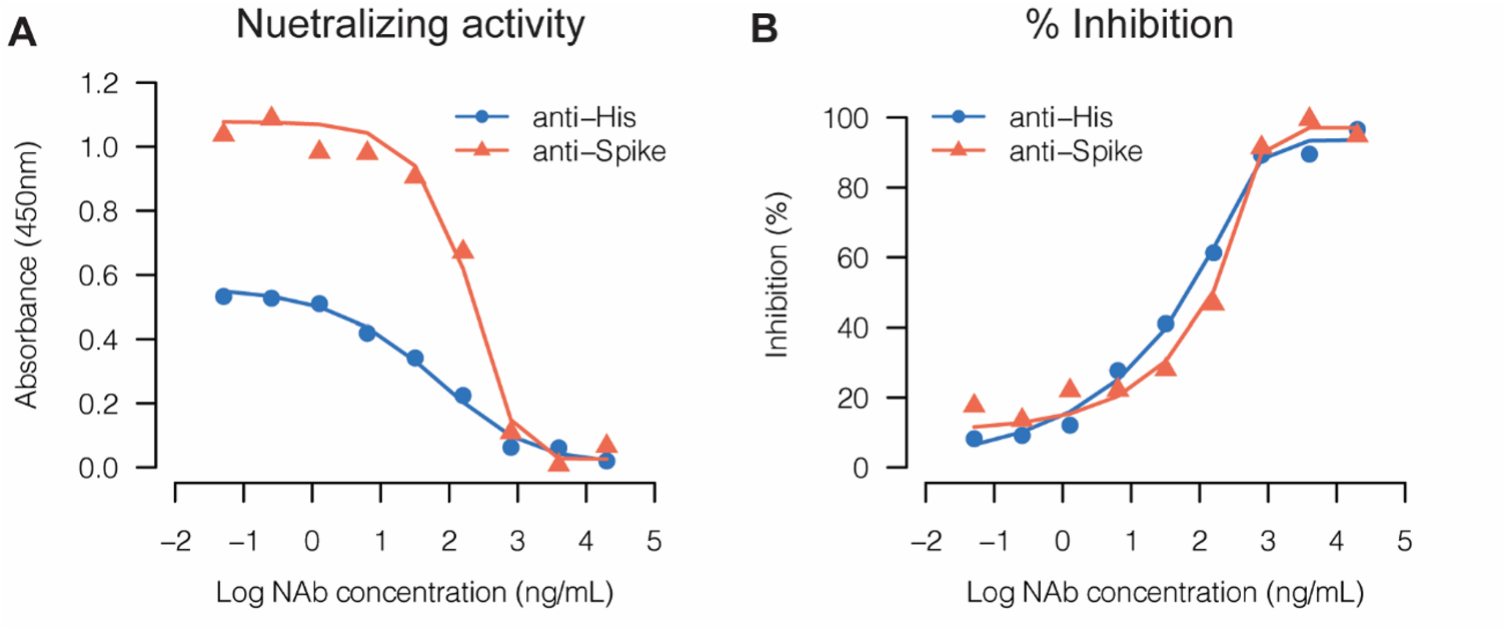
Initial evaluation of NAb detection assay set up. The competitive ELISA was performed using Delta H-RBD and MM43 NAb as a representative system. Dots represent (**A**) the mean value of absorbance at 450 nm and **B** % inhibition of 3 technical replicates

Notably, many of the previous studies have asserted that the data for evaluation of NAb from competitive ELISA correlated well with data from other types of assays employing live virus and using a recovered patient’s serum [8, 9, 22]. For our % inhibition profiling analysis, we aimed to use commercially available purified NAbs with known interactions to the specific variants for our comparison of the in-house E-RBD and purchased H-RBD. Up to that point, we had used both anti-His HRP and anti-spike RBD antibodies for detection. Before moving forward, the ELISA signals from the two different setups were evaluated using H-RBD of the Delta variant and MM43 NAb, a mouse monoclonal NAb purchased from Sino Biological that had been reported to exert broadly neutralizing activity in response to wild-type SARS-CoV-2 (Wuhan strain), Alpha, Beta, Gamma, and Delta variants but could not neutralize the Omicron variant [23, 24]. In this assay, 3 ng of Delta H-RBD was pre-incubated with MM43 NAb at various concentrations for 1 h at 37 °C (final volume of 50 μl) prior to addition into 96-well plates coated with 130 µg of ACE2-hFc per well. This protocol was adapted from Tan et al.’s [9] study, which became commercially available [9, 22]. It was clearly observed that anti-spike RBD yielded higher absorbance readings than anti-His HRP. This outcome was not surprising considering that anti-spike RBD is a polyclonal antibody; thus, there could be more than one antibody binding to one RBD. Furthermore, anti-spike RBD is not conjugated to HRP. Therefore, we used goat anti-rabbit HRP as a secondary antibody to detect anti-spike RBD and generate the ELISA signal. Goat anti-rabbit HRP is also polyclonal, meaning that it can also enhance the signal via binding to multiple epitopes on the antigen. Figure 8B demonstrates the plots of % inhibition obtained from both antibodies; the similar results shown suggest that both antibodies could be utilized in the NAb detection assay. Nonetheless, as new variants continue to emerge, it may be more suitable to apply anti-His antibody in the assay since it should detect the His-tag epitope that would be common across all recombinant RBD constructs with the same affinity. Moreover, because anti-His HRP is commonly used in many research areas, the cost of anti-His HRP is significantly cheaper than that of anti-spike RBD. Therefore, we used anti-His HRP in the next experiment to conduct a head-to-head comparison of all E-RBD and H-RBD variants produced in this study against known concentrations of NAbs that were specific to the RBD variants.

**Fig. 9:**
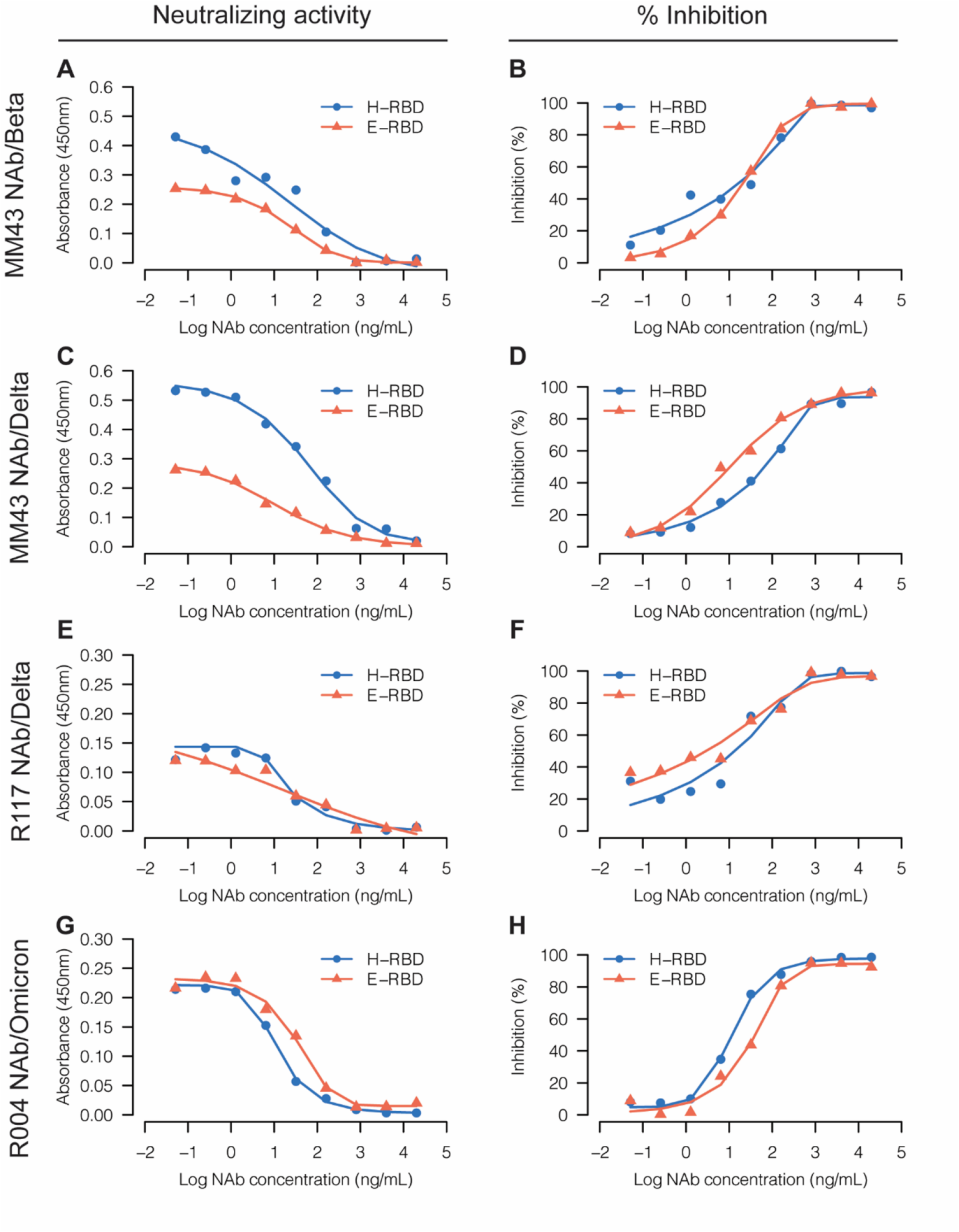
Evaluation of inhibition of interaction between ACE2-hFc and E-RBD or H-RBD by monoclonal NAbs. Competition ELISA results used the indicated antigens for three NAbs purchased from Sino Biological Inc. NAbs were serially diluted 1:5 for analysis, and bound E-RBD or H-RBD was detected with anti-His HRP. Dots represent the mean values of absorbance at 450 nm (Left panel) and % inhibition (Right panel) of three technical replicates

Figure 9 demonstrates the neutralizing activity against E-RBD and H-RBD profiling by performing a 1:5 dilution ratio of the concentrations between 20 µg/mL and 0.05 ng/mL (133—0.3 pM). In the case of MM43 NAb, which could neutralize Beta and Delta variants, it emerged that at high NAb concentrations (between 800 ng/mL and 20 µg/mL) where the ELISA absorbance values were in the plateau, E-RBD gave a very similar signal to H-RBD. However, at low concentrations of NAb (below 160 ng/mL or ~ 1 nM), E-RBD started to show a lower signal than H-RBD; furthermore, the difference increased as the NAb concentration decreased. At the lowest concentration of MM43 NAb tested (0.05 ng/mL or ~ 0.3 pM), the difference in signals from both variants of E-RBD was approximately half of H-RBD. In the case of R117 NAb, a rabbit monoclonal NAb that could neutralize Delta but not Beta or Omicron variants, we found that ELISA signals from both E-RBD and H-RBD were quite low compared to the signals from MM43 NAb. Interestingly, the signal strengths were almost the same within the range of 32 ng/mL—20 µg/mL, but they started to diverge at very low NAb concentrations. Since the ELISA signals were quite low for this assay setting, the differences at such low concentrations could fall within experimental error. Finally, the ELISA signals of the E-RBD and H-RBD Omicron variant were studied using R004 NAb, another rabbit monoclonal NAb that could neutralize the Omicron variant. As in the R117 NAb result, E-RBD and H-RBD gave comparable signal strengths. Remarkably, regardless of the differences in the observed absorbance readings, when converted into % inhibition, both E-RBD and H-RBD demonstrated comparable results across all of the investigated assays.

## Discussion

The COVID-19 pandemic has caused devastating impacts on human health, society and economy for more than 2 years. With the emergence of every new VoC, understanding the relationship between measured immunity and clinical protection from SARS-CoV-2 infection becomes ever more critical in planning the next steps in the COVID-19 vaccine program, including the duration and extent of immunity against each new variant, the requirement for booster vaccine doses, next-generation vaccine development, and efficacy studies that would require the continuation of NAb level monitoring.

In this study, E-RBD was purified from inclusion bodies using an on-column refolding technique. From 200 mL of cell culture in a 1-L shake flask, we successfully obtained E-RBDs of the Beta, Delta, and Omicron variants from a simple on-column refolding IMAC method. Nevertheless, the yields were quite low (0.4—1 mg/L), which could have been due to incomplete extraction of the protein, as a large amount of RBD was still detected in the fraction containing cell debris (Fig. 5A). He et al. [17] reported the ability to obtain 13.3 mg/L RBD from *E. coli* by flask culture from inclusion bodies, in comparison to producing 5 mg/L RBD from mammalian cells (HEK-293 T) via cell culture [17]. Even though purified RBD from the Beta variant revealed slightly lower purity compared to other variants (70% vs. 90%), this factor should not affect the NAb detection assay since the format of this assay involves a specific interaction between RBD and ACE2. Moreover, as indicated by the WB results in Fig. 5, any contaminants present in the Coomassie-stained gels in Fig. 4 did not cross-react with anti-His-HRP used in this study, as only a single band was observed in each lane.

Previous studies have already confirmed that competitive ELISA based on the interaction between RBD and ACE2 correlates well with the standard neutralization assay [8, 9]; in addition, other research findings have indicated that antibodies in human serum could recognize RBD produced in *E. coli* [16, 18]. Accordingly, this study aimed to use well-characterized, commercially available NAb to compare differences between E-RBD and H-RBD binding to ACE2. The use of purified NAbs also allowed us to evaluate the results with known concentrations of the NAbs. Specifically, we investigated the effect of glycosylation on the performance of E-RBD using the direct binding ELISA detected by anti-His HRP and anti-spike RBD. The result was very encouraging: E-RBD gave signals that were almost identical to the ones characteristic of the commercial H-RBD. Because our study used RBDs derived from amino acid residues 319–541 of the S protein, only N331 and N343 glycosites were involved. According to the characterization of RBD containing amino acid 330–583 of S protein from the strain Wuhan-Hu-1 (GenBank Acc. No. 045512.2), which possesses the same glycosites as our RBD, even with the absence of glycosylation, FT-IR spectroscopy only showed a slight structural alteration. Circular dichroism results suggested that the major β-sheet content of E-RBD was almost unaltered; in addition, fluorescence spectroscopy revealed that the tertiary structure of E-RBD was only slightly changed [17]. Moreover, previous studies have demonstrated that five glycosites located near or on RBD, specifically N165, N234, N282, N331, and N343, affected the binding of RBD to the ACE2 receptor. The effect of N-linked glycosylation may be more pronounced when the glycosylation is present in the full-length S protein in light of the fact that the glycosylation at these sites can regulate the RBD’s conformation dynamic (referred to as RBD being in the “up” or “down” states) or shield the binding sites of S protein [25]. When the RBD is expressed alone, it is present in the monomeric form; therefore, it is always accessible to ACE2.

In the case of both the Beta and Delta variants, lower ELISA signal intensities were observed from interactions between MM43 NAb and E-RBD than H-RBD RBD *(Fig. 9A, C). Looking specifically at the results from the Delta variant *(Fig. 9C, E) reveals that both MM43 NAb and R117 NAb could neutralize the Delta variant. That said, the 2 NAbs showed different signal strengths between E-RBD and H-RBD: (1) MM43 NAb gave lower signals in response to E-RBD than H-RBD, while R117 NAb showed comparable signals, and (2) MM43 NAb yielded higher signal intensities than R117 NAb (the highest OD_450_ from MM43 NAb was approximately 0.5 in comparison to only 0.14 from R117 NAb). In all, it appeared that the difference in signal strength between E-RBD and H-RBD might have depended upon the NAb itself rather than the RBD. Previous studies have also demonstrated the effect of glycosylation at some sites on the sensitivity of viruses to neutralizing antibodies; for instance, the N149Q, N331Q, and N1173Q mutations in S protein dramatically increased sensitivity to convalescent sera [25]. As these NAbs could bind at different epitopes, we hypothesized that MM43 might bind to an epitope near the N331 glycosite; hence, the influence of glycan on this NAb likely affects the ELISA signals. Nonetheless, % inhibition data generated from E-RBD and H-RBD demonstrated comparable results, suggesting that E-RBD could be applied in the development of a NAb detection assay, as the effect of glycosylation became negligible when % inhibition was considered.

In this study, we demonstrated the possibility of using *E. coli* as an expression system for the development of a NAb detection assay. As the battle between humans and SARS-CoV-2 seems far from over, NAb detection based on an ELISA format could provide a powerful tool to help accelerate the research and management of COVID-19 in many areas, such as the evaluation of vaccine efficacy, development of NAb for therapeutics, and serology surveillance of the community [22]. Since the commercially available test kits for NAb detection are at this point very expensive, we hope that this research will contribute to the development of NAb detection immunoassays that would employ *E. coli* as the SARS-CoV-2 spike RBD expression system. This system can be modified easily and set up in any molecular biology laboratory, offering a significant advantage in managing COVID-19 in low-income countries where the cost of commercial test kits can be obstacle and local variants are likely to emerge. On another note, a few previous studies also attempted to use *E. coli* as an expression system for ACE2 [26, 27]. It remains to be investigated if the combination of hACE2 and E-RBD produced from *E. coli* could be used for the development of a NAb detection assay. This knowledge will be particularly useful in the present and near future. As new variants emerge and new generations of vaccinations are deployed, anti-SARS-CoV-2 antibody assays will be useful for the reliable, scalable, and cost-effective quantification of the extent of immunity conferred to populations.

## Supplementary Information

Below is the link to the electronic supplementary material.

Supplementary file1 (DOCX 30 kb)

## Data Availability

All data generated or analyzed during this study are included in this article (and its supplementary information) or are available from the corresponding authors on reasonable request.
