# Supplementary material for: Utilization of Receptor-Binding Domain of SARS-CoV-2 Spike Protein Expressed in Escherichia coli for the Development of Neutralizing Antibody Assay
Source: Mol Biotechnol. 2022 Sep 14;65(4):598–611. doi: 10.1007/s12033-022-00563-4 (PMC9472194; doi:10.1007/s12033-022-00563-4)
Supplement: Supplementary file 1 — Supplementary file1 (DOCX 30 kb) [file 12033_2022_563_MOESM1_ESM.docx]

**Supplementary Data**

**Densitometry analysis**

The protocol was adapted from Alonso Villela et al.’s (2020) [1] proposed protocol for image analysis of electrophoresis gels to quantify unknown proteins based on normalization using the protein markers in SDS-PAGE gels. In this study, densitometry was used to quantify the RBD yield of the insoluble fraction from each induction condition relative to the RBD yield from protein expression at 25 °C, 2 hours, and 0.1 mM IPTG and calculated per 100 mL of culture (Y_Relative_). First, the OD_600_ of each cell culture was measured and used to calculate harvested volume (V_Harvested_) before being normalized in 300 µL PBS to OD_600_ = 25. After sonication, samples were collected as soluble and insoluble fractions as described in the materials and methods section. For Coomassie analysis, 40 µL of normalized samples were loaded. The intensity of all protein bands was quantified by Image Lab software (Bio-Rad). Since variation was possible during the staining procedure, the intensity of the 25-kDa protein marker band from each gel (I_M_) was used for normalization with the reference marker band, which was obtained from the gel containing samples from the expression at 25 °C, 0.1 mM IPTG for 2 hours.

The marker ratio was calculated as shown in Equation 1,

$M_{ratio} = \frac{I_{M,sam}}{I_{M,ref}}$ (1)

where the marker ratio ($M_{ratio}$) represents the ratio of the 25-kDa marker band intensity from each condition ($I_{M,sam}$) to the 25-kDa marker band intensity of the reference condition ($I_{M,Ref}$).

The intensity of the RBD band from each gel was then calculated per 100 mL culture volume using Equation 2, as follow:

$I_{RBD, 100 mL} = \frac{M_{ratio}\times I_{RBD}\times100}{V_{Harvested}}$ (2)

where $I_{RBD, 100 mL}$ denoted the RBD band intensity calculated for 100 mL culture volume and compared with the reference condition, $I_{RBD}$ indicates the RBD band intensity at each condition, and $V_{Harvested}$ represents the normalized culture volume collected for sonication.

Finally, the relative yield of RBD produced from each condition ($Y_{Relative}$) was calculated as shown in Equation 3:

$Y_{Relative} =\frac{I_{RBD, 100 mL}}{I_{RBD,Ref}}$ (3)

where $Y_{Relative}$ signifies the ratio of the calculated RBD band intensity from each condition at 100 mL cell culture volume to the calculated RBD band intensity from the reference condition at 100 mL cell culture volume.

**Reference:** Alonso Villela, S. M.; Kraiem, H.; Bouhaouala-Zahar, B.; Bideaux, C.; Aceves Lara, C. A.; Fillaudeau, L., A protocol for recombinant protein quantification by densitometry. *Microbiologyopen* **2020,** 9, (6), 1175-1182.

**Table S1** Band intensity from each induction condition for densitometry analysis

| Condition | Strain | IPTG (mM) | Incubation time | 25-kDa marker band intensity  (I_M_) | Marker ratio  (M_Ratio_) | RBD band intensity  (I_RBD_) | OD_600_ | Harvested cell culture volume (mL) (V_Harvested_) | Calculated RBD band intensity at 100 mL cell culture volume  (I_RBD,100mL_) | Relative yield of RBD  (Y_Relative_) |
| --- | --- | --- | --- | --- | --- | --- | --- | --- | --- | --- |
| 25°C  2 Hours | BL | 0.1 | 2 | 40395.869  (I_M,Ref_) | 1 | 16429.229 | 1.212 | 6.2 | 264987.5645 | 1 |
|  | BL | 0.5 | 2 |  |  | 17991.3 | 1.23 | 6.1 | 294939.3443 | 1.113030888 |
|  | SH | 0.1 | 2 |  |  | 24266.141 | 1.272 | 5.1 | 475806.6863 | 1.795581189 |
|  | SH | 0.5 | 2 |  |  | 26626.768 | 1.212 | 6.2 | 429464 | 1.620694921 |
| 25°C  4 Hours | BL | 0.1 | 4 | 45938.354 | 1.137204252 | 9121.681 | 1.3 | 5.8 | 178848.5245 | 0.674931765 |
|  | BL | 0.5 | 4 |  |  | 9384.853 | 1.4 | 5.8 | 184008.53 | 0.694404397 |
|  | SH | 0.1 | 4 |  |  | 40312.801 | 1.455 | 5.155 | 889309.1895 | 3.356041221 |
|  | SH | 0.5 | 4 |  |  | 47320.801 | 1.3 | 5.8 | 927817.5193 | 3.501362492 |

**Table S1** Band intensity from each induction condition for densitometry analysis (Cont’d)

| Condition | Strain | IPTG (mM) | Incubation time | 25-kDa marker band intensity  (I_M_) | Marker ratio  (M_Ratio_) | RBD band intensity  (I_RBD_) | OD_600_ | Harvested cell culture volume (mL) (V_Harvested_) | Calculated RBD band intensity at 100 mL cell culture volume  (I_RBD,100mL_) | Relative yield of RBD  (Y_Relative_) |
| --- | --- | --- | --- | --- | --- | --- | --- | --- | --- | --- |
| 30°C  2 Hours | BL | 0.1 | 2 | 45718.153 | 1.131753175 | 34695.688 | 0.935 | 8.02 | 489612.9059 | 1.847682576 |
|  | BL | 0.5 | 2 |  |  | 38023.638 | 1.029 | 7.2875 | 590509.407 | 2.228441957 |
|  | SH | 0.1 | 2 |  |  | 42832.236 | 1.324 | 5.665 | 855702.0139 | 3.229215739 |
|  | SH | 0.5 | 2 |  |  | 52021.6 | 1.154 | 6.5 | 905778.6303 | 3.418192971 |
| 30°C  4 Hours | BL | 0.1 | 4 | 39553.718 | 0.979152547 | 28208.011 | 1.174 | 6.49 | 425576.9769 | 1.606026221 |
|  | BL | 0.5 | 4 |  |  | 29186.153 | 1.314 | 5.7 | 501363.0883 | 1.892024968 |
|  | SH | 0.1 | 4 |  |  | 44517.215 | 1.138 | 6.6 | 660441.5823 | 2.492349343 |
|  | SH | 0.5 | 4 |  |  | 45959.337 | 1.105 | 7.5 | 600016.0248 | 2.26431767 |

| Condition | Strain | IPTG (mM) | Incubation time | 25-kDa marker band intensity  (I_M_) | Marker ratio  (M_Ratio_) | RBD band intensity  (I_RBD_) | OD_600_ | Harvested cell culture volume (mL) (V_Harvested_) | Calculated RBD band intensity at 100 mL cell culture volume  (I_RBD,100mL_) | Relative yield of RBD  (Y_Relative_) |
| --- | --- | --- | --- | --- | --- | --- | --- | --- | --- | --- |
| 37°C  2 Hours | BL | 0.1 | 2 | 31077.655 | 0.769327552 | 54063.023 | 1.53 | 4.9 | 848819.8597 | 3.203244127 |
|  | BL | 0.5 | 2 |  |  | 52933.68 | 1.475 | 5.1 | 798496.8322 | 3.013336998 |
|  | SH | 0.1 | 2 |  |  | 53459.245 | 1.02 | 7.35 | 559560.1371 | 2.111646779 |
|  | SH | 0.5 | 2 |  |  | 57118.408 | 0.985 | 7.6 | 578194.2762 | 2.181967585 |
| 37°C  4 Hours | BL | 0.1 | 4 | 32400.362 | 0.802071172 | 39337.768 | 1.64 | 4.6 | 685906.2976 | 2.588447118 |
|  | BL | 0.5 | 4 |  |  | 44988.86 | 1.6 | 4.7 | 767750.3761 | 2.897307191 |
|  | SH | 0.1 | 4 |  |  | 45334.366 | 0.836 | 9 | 404015.4231 | 1.524658049 |
|  | SH | 0.5 | 4 |  |  | 47562.881 | 0.782 | 9.6 | 397383.497 | 1.499630738 |

**Table S1** Band intensity from each induction condition for densitometry analysis (Cont’d)

BL and SH abbreviate *E. coli BL21(DE3) and SHuffle* strains, respectively.
